# Red Meat and Poultry Intakes and Risk of Total and Cause-Specific Mortality: Results from Cohort Studies of Chinese Adults in Shanghai

**DOI:** 10.1371/journal.pone.0056963

**Published:** 2013-02-22

**Authors:** Yumie Takata, Xiao-Ou Shu, Yu-Tang Gao, Honglan Li, Xianglan Zhang, Jing Gao, Hui Cai, Gong Yang, Yong-Bing Xiang, Wei Zheng

**Affiliations:** 1 Vanderbilt Epidemiology Center, Vanderbilt University, Nashville, Tennessee, United States of America; 2 Shanghai Cancer Institute, Department of Epidemiology, Shanghai, China; Brigham and Women’s Hospital and Harvard Medical School, United States of America

## Abstract

Most previous studies of meat intake and total or cause-specific mortality were conducted in North America, whereas studies in other areas have been limited and reported inconsistent results. This study investigated the association of red meat or poultry intake with risk of total and cause-specific mortality, including cancer and cardiovascular disease (CVD), in two large population-based prospective cohort studies of 134,290 Chinese adult women and men in Shanghai. Meat intakes were assessed through validated food frequency questionnaires administered in person at baseline. Vital status and dates and causes of deaths were ascertained through annual linkage to the Shanghai Vital Statistics Registry and Shanghai Cancer Registry databases and home visits every 2–3 years. Cox regression was used to calculate hazard ratios (HRs) and 95% confidence intervals (CIs) for the risk of death associated with quintiles of meat intake. During 803,265 person-years of follow up for women and 334,281 person-years of follow up for men, a total of 4,210 deaths in women and 2,733 deaths in men accrued. The median intakes of red meat were 43 g/day among women and 54 g/day among men, and pork constituted at least 95% of total meat intake for both women and men. Red meat intake was associated with increased total mortality among men, but not among women; the HR (95% CI) comparing the highest with the lowest quintiles were 1.18 (1.02–1.35) and 0.92 (0.82–1.03), respectively. This sex difference was statistically significant (*P* = 0.01). Red meat intake was associated with increased risk of ischemic heart disease mortality (HR = 1.41, 95% CI = 1.05–1.89) and with decreased risk of hemorrhagic stroke mortality (HR = 0.62, 95% CI = 0.45–0.87). There were suggestive inverse associations of poultry intake with risk of total and all-CVD mortality among men, but not among women. Further investigations are needed to elucidate the sex-specific associations between red meat intake and mortality.

## Introduction

Red meat intake, especially processed meat, has been positively associated with risk of total and cause-specific mortality in some studies [1]–[3], but not all [4]–[6]. It has been hypothesized that the positive association of red meat intake with the risk of chronic diseases may be attributed to high saturated fatty acid and heme iron content or carcinogens, including heterocyclic amines and *N*-nitroso compounds [7]–[9]. In contrast, poultry is often considered one of the healthier alternatives to red meat and has been inversely associated with the risk of total or cardiovascular disease (CVD)-related mortality in a few studies [4], [10]. Inconsistent findings on the association between meat intake and mortality reported in previous epidemiologic studies may be partly explained by different methods of processing and cooking meats; processed meat intake has been more strongly associated with the risk of mortality than unprocessed meat intake [2]. Unlike in North American and European countries, in Shanghai, China, consuming processed meats and grilled meats is uncommon and pork is the predominant component of total red meat intake (>95%) rather than beef. In addition, in China meats are often stewed or stir-fried with vegetables, and blood is typically drained from meat before consumption. Hence, meats in China may have lower amounts of heme iron than meat consumed in Western countries. Therefore, it is possible that the association between meat intake and mortality outcomes in the Chinese population may differ from that observed in studies conducted in North American and European countries. In this report, we investigated the association of red meat and poultry intakes with the risk of mortality from all causes and specific causes, including cancer and CVD, by using data from two prospective cohort studies of 134,290 adult women and men in Shanghai, China.

## Materials and Methods

### Study Population

The studies’ protocols were approved by the Institutional Review Boards of all institutes involved in the study, including Vanderbilt University, the Shanghai Cancer Institute, and the US National Cancer Institute. Written informed consent was obtained from all participants prior to interview. This report includes participants of the Shanghai Women’s Health Study (SWHS) and Shanghai Men’s Health Study (SMHS), two population-based, prospective cohort studies. Through home visits, the SWHS recruited 74,941 women from 1997 to 2000 who were residing in urban areas of Shanghai and were aged 40 to 70 years, and the SMHS recruited 61,483 men from 2002 to 2006 who were aged 40 to 74 years [11], [12].

### Dietary Assessment

Intakes of red meat and poultry, as well as other food items, were assessed using quantitative food frequency questionnaires (FFQ), which were developed and validated separately for women [13] and men [14]. The correlation coefficients between multiple 24-hour dietary recalls and the FFQs for red meat were 0.52 for women and 0.45 for men and for poultry were 0.48 for women and 0.35 for men. Both FFQs used the same format and included the same questions for meat intakes. Red meat intake was based on nine questions, which collected intake information on pork chops; pork ribs; pig’s feet; regular fresh pork; lean fresh pork; mixed fresh pork; pig, cow, and sheep liver; organ meat, including heart, brain, tongue, tripe, and intestine; and beef and lamb. Poultry intake was based on two questions on intakes of chicken, duck, and goose. Both FFQs assessed typical intake during the year before study enrollment by asking about the frequency (five categories ranging from never to every day) and the amount of consumption in *liang* (1 *liang* is equivalent to 50 grams). The Chinese Food Composition Tables were used to calculate intakes of total calories and nutrients [15].

### Cohort Follow Up and Death Ascertainment

Participants’ residential identification number, name, and address were linked to the Shanghai Vital Statistics Registry and Shanghai Cancer Registry databases annually, and in-person visits to participants’ homes were made every 2–3 years. The response rates were 99.8%, 98.7%, 96.7%, and 92.3%, respectively, for the first to fourth in-person, follow-up surveys in the SWHS, and 97.6% and 93.6%, respectively, for the first and second in-person, follow-up surveys in the SMHS. All possible matches identified through the linkage were verified by home visits. Follow up for mortality is nearly complete. The underlying cause of death reported on the death certificate was considered to be the cause of death in the analyses and was coded according to the International Classification of Diseases, Ninth Revision (ICD-9) [16]. Causes of death were first grouped into major diseases, including CVD (ICD-9 = 390–459) and cancer (ICD-9 = 140–208). Deaths due to CVD or cancer were further divided into the top three CVD-related causes and the top four cancer-related causes. For CVD, this included ischemic heart disease (ICD-9 = 410–414), hemorrhagic stroke (ICD-9 = 430–431), and ischemic stroke (ICD-9 = 433–435). For cancer, this included lung cancer (ICD = 162), stomach cancer (ICD = 151), colorectal cancer (ICD = 153–154), and liver cancer (ICD = 155). We also grouped cancers by smoking- and non-smoking-related cancers based on a report from the International Agency for Research on Cancer [17]. Since the number of deaths due to diabetes was relatively small (n = 93) in the SMHS due to the shorter follow-up time, diabetes mortality was not included in our analyses.

### Statistical Analysis

All statistical analyses were conducted by using SAS, version 9.3 (Cary, NC). In the current analyses, we excluded participants with a prior history of cancer at baseline (1,579 women; no men were excluded, since this was an exclusion criterion for participation in the SMHS), those who reported a total caloric intake outside the range of 500 to 4,000 kcal per day (50 women and 91 men), and those with no follow up (5 women and 14 men). We further excluded participants who died during the first year of observation (145 deaths in women and 249 deaths in men) to minimize the possibility of reverse causality, and we excluded one male participant who did not answer all questions regarding smoking history. Very few participants had missing data for covariates; we replaced missing covariate data with the most common category [e.g., education (0.65%), occupation (0.05%), income (0.11%), BMI (0.03%), and pack-years of smoking among men (0.002%)] in each of the cohorts. The final analyses included 73,162 women and 61,128 men.

Characteristics of the study population were described according to quintiles of red meat intake after adjusting for age at baseline, separately for women and men. Likewise, intakes of meats, fish, fruits, and vegetables were described after further adjusting for total caloric intake. Sex-specific quintiles of intakes of red meat (sum of pork and beef/lamb intakes), poultry, and pork were created based on the distribution among all cohort members at baseline. The median value of each quintile was assigned and used to test for linear trend. The risk of mortality was assessed for total (all causes) and cause-specific mortality as described above. Cox proportional hazards regression with age as the time scale was performed to calculate hazard ratios (HRs) and the corresponding 95% confidence intervals (CIs) for each quintile of intake by using the lowest quintile as the reference. HRs and 95% CIs were derived separately for women and men. Entry time was defined as age at one year after study enrollment, and exit time was defined as age at death or censoring at December 31, 2010 (the date of the latest record linkage to verified data), whichever came first. Variables that were associated with red meat intake, poultry intake, or total mortality in our study populations were adjusted for in the final model as covariates. These included age at baseline (continuous), total caloric intake (continuous), smoking history (pack-years of smoking for men and ever/never smoking for women), income (four categories), occupation (three categories), education (four categories), comorbidity index (three categories based on the number of existing chronic diseases [18]), physical activity (categories based on MET-hours per day), total vegetable intake (quintiles), total fruit intake (quintiles), and regular alcohol consumption for men (three categories). We also tested non-linear associations of red meat intake with total and cause-specific mortality by using restricted cubic spline Cox regression models placing knots at the 5^th^, 35^th^, 65^th^, and 95^th^ percentiles of intake. Results for women and men were combined to obtain summary risk estimates using the meta-analysis approach. Risk estimates from a random-effects model are presented when the test for heterogeneity was significant; risk estimates from a fixed-effects model are presented when significant heterogeneity was not present [19], [20]. Tests for heterogeneity (Cochran’s *Q* statistics and its *P*-value) [21] were used to assess sex differences for total and cause-specific mortality risk when comparing the highest quintiles of intake with the lowest.

Stratified analyses were conducted by income (lower than 1,000 *yuan* per capita or higher for men and lower than 20,000 *yuan* per household or higher for women), education [low (up to and including middle school) or high (high school and higher)], the existence of comorbidity (none or any), history of hypertension at baseline (yes or no), history of diabetes at baseline (yes or no), regular exercise (yes or no), and BMI (<25 kg/m^2^ or ≥25 kg/m^2^). Among men, stratified analyses by smoking status (never, past, or current smokers) and by regular alcohol consumption (none, <2 drinks/day, or ≥2 drinks/day) were also conducted. Interaction effects between meat intake and selected characteristics were tested by including the main effects and the product terms in a model and assessing the *P*-value of the product term. Sensitivity analyses were conducted by excluding the first two years of observation and related deaths (216 deaths in women and 397 deaths in men during the second year of observation).

## Results

There were 4,210 deaths after a median follow up of 11.2 years in the SWHS and 2,733 deaths after a median follow up of 5.5 years in the SMHS, excluding the first year of follow up (**Table 1**). The person-years of follow up for women and men were 803,265 and 334,281, respectively. In both cohorts, more older participants were classified in the lower meat consumption categories than were younger participants. Higher red meat intake tended to be related to higher socioeconomic status among women, but not among men. Smoking was more common among those in the highest quintile of red meat intake than among those in the lowest among men. Total caloric intake tended to be higher among those who consumed more red meat than among those who consumed less. Pork intake constituted at least 95% of total red meat intake in both cohorts (97% in women and 95% in men) and beef/lamb intake was very small (median intake: 1.3 g/day in women and 2.6 g/day in men).

**Table 1:** Characteristics of the study populations of the Shanghai Women’s Health Study and Shanghai Men’s Health Study by red meat intake.*

	Women		Men	
Red meat intake	Quintile 1	Quintile 5	Quintile 1	Quintile 5
Number of participants	14,633	14,632	12,226	12,225
Age at baseline (years)	55.2±9.5	50.5±8.2	58.2±10.3	52.7±8.7
Education				
Elementary school or less	24.3%	18.3%	7.7%	6.4%
Middle school	37.7%	38.1%	33.1%	34.4%
High school	26.9%	28.4%	36.6%	36.5%
Professional education/college or higher	11.2%	15.1%	22.5%	22.7%
Income**				
Low	19.1%	16.0%	13.8%	13.5%
Lower middle	38.7%	38.1%	41.3%	43.1%
Upper middle	25.7%	28.6%	35.0%	33.6%
High	16.5%	17.4%	9.8%	9.7%
Occupation				
Professional workers	25.7%	29.9%	26.1%	25.5%
Clerical workers	22.1%	20.5%	21.8%	21.9%
Manual laborers	52.2%	49.6%	52.1%	52.6%
Smoking				
Ever smoked	3.2%	3.1%	67.9%	73.4%
Smoking (pack-years)***	–	–	23.9±0.18	26.8±0.16
Alcohol consumption				
Ever consumed	2.1%	2.8%	30.9%	39.1%
Drinks (day)***	–	–	0.77±0.02	1.10±0.02
Regular tea consumption	27.6%	31.6%	63.2%	70.7%
BMI (kg/m^2^)	24.0±0.03	24.2±0.03	23.6±0.03	23.9±0.03
Vitamin supplement use†	27.6%	31.6%	14.0%	13.5%
Physical activity (MET-hours)	2.01±0.03	2.04±0.03	0.99±0.02	1.03±0.02
Total caloric intake (kcal/day)	1,454±2.9	1,991±2.9	1,647±3.9	2,263±3.9
Red meat intake (g/day)‡	16.5±0.15	103.4±0.15	21.4±0.19	126.0±0.20
Poultry intake (g/day)‡	11.9±0.15	19.9±0.15	11.9±0.17	22.3±0.18
Fish intake (g/day)‡	51.6±0.36	50.8±0.37	50.3±0.41	57.9±0.43
Vegetable intake (g/day)‡	313.9±1.34	288.7±1.38	345.2±1.68	350.7±1.73
Fruit intake (g/day)‡	281.9±1.44	241.5±1.49	158.0±1.14	136.9±1.17

* Adjusted for age at baseline survey, except for age and food intakes, and shown as mean ± standard errors; age shown as mean standard ± deviation.

** Cut-points for income are as follows: for women: low, <10,000 *yuan* per household; lower middle, 10,000–19,999 *yuan*; upper middle, ≥20,000–29,999 *yuan*; and high, ≥30,000 *yuan* and for men: low, <500 *yuan* per capita; lower middle, 500–999 *yuan*; upper middle, 1,000–1,999 *yuan*; and high, ≥2,000 *yuan*.

*** Only smokers or alcohol consumers were included.

† Any individual vitamin A, C, D, A/D or E supplement or multivitamins.

‡ Adjusted for age and total caloric intake.

Red meat intake was positively associated with total mortality among men, whereas no association was found among women; the HRs (95% CIs) comparing the highest quintile of intake with the lowest were 1.18 (1.02–1.35) for men and 0.92 (0.82–1.03) for women (**Table 2**). A significant linear trend was observed among men (*P*-trend = 0.02), but not among women (*P*-trend = 0.54). This difference in the association by sex was significant (*P*-heterogeneity = 0.01). Similar patterns of association were observed for pork intake. Red meat and pork intakes were not significantly associated with all-cancer or all-CVD mortality in either women or men. Among men, we observed a suggestive inverse association of poultry intake with the risk of total (HR = 0.90, 95% CI = 0.79–1.02) and all-CVD mortality (HR = 0.81, 95% CI = 0.65–1.02). Especially for all-CVD mortality, the risk for the second to fourth quintiles were all significantly lower than the first quintile, although there was no significant linear trend (*P*-trend = 0.13). Among women, poultry intake was not significantly associated with total, all-cancer, or all-CVD mortality.

**Table 2:** Risk of total, all-cancer, and all-CVD mortality by quintiles of meat intake.*

			Red meat				Poultry				Pork				
	Quintile		Deaths	HR	95%	CI	Deaths	HR	95%	CI	Deaths	HR	95%	CI	
Women															
Total															
	1		1282	Reference			1271	Reference			1224	Reference			
	2		858	0.87	0.80,	0.96	926	0.91	0.84,	1.00	891	0.92	0.84,	1.00	
	3		777	0.93	0.85,	1.03	750	0.94	0.86,	1.04	796	0.98	0.89,	1.07	
	4		704	0.98	0.89,	1.09	675	0.96	0.86,	1.06	699	0.98	0.88,	1.08	
	5		589	0.92	0.82,	1.03	588	0.98	0.88,	1.09	600	0.93	0.84,	1.05	
	*P*-trend				0.54				0.86				0.46		
All cancers															
	1		478	Reference			470	Reference			457	Reference			
	2		364	0.90	0.78,	1.04	393	0.93	0.81,	1.07	381	0.96	0.83,	1.10	
	3		333	0.90	0.78,	1.05	319	0.89	0.77,	1.04	345	0.96	0.83,	1.11	
	4		328	0.98	0.85,	1.15	320	0.95	0.81,	1.11	315	0.95	0.82,	1.11	
	5		294	0.92	0.78,	1.09	295	0.96	0.82,	1.13	299	0.94	0.79,	1.11	
	*P*-trend				0.62				0.96				0.51		
All CVD															
	1		439	Reference			424	Reference			409	Reference			
	2		267	0.84	0.72,	0.99	271	0.88	0.75,	1.03	292	0.96	0.82,	1.12	
	3		231	0.91	0.77,	1.07	244	1.08	0.92,	1.28	224	0.92	0.77,	1.09	
	4		191	0.90	0.75,	1.09	193	1.02	0.85,	1.23	207	0.99	0.83,	1.19	
	5		160	0.89	0.72,	1.09	156	1.03	0.84,	1.26	156	0.87	0.71,	1.08	
	*P*-trend				0.37				0.47				0.30		
Men															
Total															
		1	812	Reference			707	Reference			787	Reference			
		2	578	0.95	0.85,	1.06	567	0.83	0.74,	0.93	568	0.93	0.83,		1.04
		3	487	0.97	0.87,	1.10	495	0.85	0.75,	0.95	495	0.96	0.86,		1.08
		4	427	0.99	0.87,	1.12	487	0.83	0.73,	0.94	435	0.99	0.88,		1.13
		5	429	1.18	1.02,	1.35	477	0.90	0.79,	1.02	448	1.16	1.02,		1.33
		*P*-trend			0.02				0.50				0.02		
All cancers															
		1	331	Reference			280	Reference			325	Reference			
		2	257	0.98	0.83,	1.16	242	0.85	0.71,	1.01	257	0.97	0.82,		1.14
		3	230	1.02	0.85,	1.21	239	0.94	0.78,	1.12	222	0.96	0.80,		1.14
		4	206	1.01	0.84,	1.22	248	0.95	0.79,	1.13	217	1.05	0.87,		1.26
		5	211	1.17	0.95,	1.44	226	0.90	0.75,	1.10	214	1.11	0.91,		1.36
		*P*-trend			0.12				0.72				0.21		
All CVDs															
		1	288	Reference			260	Reference			277	Reference			
		2	196	0.95	0.79,	1.14	194	0.80	0.66,	0.97	191	0.93	0.77,		1.13
		3	144	0.90	0.73,	1.11	149	0.75	0.61,	0.92	147	0.89	0.72,		1.10
		4	122	0.91	0.73,	1.15	133	0.67	0.54,	0.84	125	0.93	0.74,		1.16
		5	125	1.15	0.90,	1.48	139	0.81	0.65,	1.02	135	1.18	0.93,		1.50
		*P*-trend			0.41				0.13				0.25		
Women+Men†															
Total															
		1	2094	Reference			1978	Reference			2011	Reference			
		2	1436	0.90	0.84,	0.97	1493	0.88	0.82,	0.94	1459	0.92	0.86,		0.99
		3	1264	0.95	0.88,	1.02	1245	0.90	0.84,	0.97	1291	0.97	0.90,		1.04
		4	1131	0.98	0.91,	1.06	1162	0.90	0.83,	0.98	1134	0.98	0.91,		1.06
		5	1018	1.04	0.82,	1.32	1065	0.95	0.87,	1.03	1048	1.04	0.84,		1.29
		*P*-trend			0.36				0.75				0.36		
			*Q* = 7.09, *P*-heterogeneity = 0.01				*Q* = 0.95, *P*-heterogeneity = 0.33				*Q* = 5.88, *P*-heterogeneity = 0.02				
All cancers															
		1	809	Reference			750	Reference			782	Reference			
		2	621	0.93	0.84,	1.04	635	0.90	0.81,	1.00	638	0.96	0.86,		1.07
		3	563	0.95	0.85,	1.06	558	0.91	0.81,	1.02	567	0.96	0.86,		1.07
		4	534	1.00	0.88,	1.12	568	0.95	0.84,	1.06	532	0.99	0.88,		1.12
		5	505	1.01	0.89,	1.16	521	0.94	0.83,	1.06	513	1.01	0.88,		1.14
		*P*-trend			0.20				0.78				0.98		
			*Q* = 3.18, *P*-heterogeneity = 0.07				*Q* = 0.24, *P*-heterogeneity = 0.62				*Q* = 1.57, *P*-heterogeneity = 0.21				
All CVD															
		1	727	Reference			684	Reference			686	Reference			
		2	463	0.89	0.79,	1.00	465	0.85	0.75,	0.96	483	0.95	0.84,		1.07
		3	375	0.90	0.79,	1.03	393	0.91	0.63,	1.30	371	0.91	0.79,		1.03
		4	313	0.91	0.79,	1.05	326	0.83	0.56,	1.25	332	0.97	0.84,		1.11
		5	285	0.99	0.84,	1.16	295	0.93	0.79,	1.08	291	1.00	0.85,		1.17
		*P*-trend			>.99				0.49				>.99		
			*Q* = 2.47, *P*-heterogeneity = 0.12				*Q* = 2.30, *P*-heterogeneity = 0.13				*Q* = 3.38, *P*-heterogeneity = 0.07				

* Adjusted for age at baseline, total caloric intake, income, occupation, education, comorbidity index, physical activity level, total vegetable intake, total fruit intake, fish intake, and red meat or poultry intake where appropriate, smoking history (ever/never smoking for women and pack-years of smoking for men), and alcohol consumption (for men only). Among women, median intakes for the first to fifth quintiles were 15.0, 29.9, 43.4, 60.1, and 94.8 g/day for red meat; 1.4, 5.4, 10.3, 17.4, and 33.8 g/day for poultry; and 13.4, 27.8, 40.8, 56.6, and 89.2 g/day for pork. Among men, the median intakes for the first to fifth quintiles were 20.0, 37.7, 53.9, 74.6, and 114.9 g/day for red meat; 0.9, 5.4, 10.9, 18.6, and 37.9 g/day for poultry; and 17.2, 34.2, 49.4, 68.5, and 106.1 g/day for pork.

† Combined risk estimates derived from a random-effects meta-analysis are presented when the test for heterogeneity was significant; combined risk estimates from a fixed-effects meta-analysis are presented when significant heterogeneity was not present. *Q* statistics and *P*-heterogeneity for risk estimates comparing the highest quintiles of intake with the lowest are shown.

For specific CVD mortality, red meat intake was positively associated with ischemic heart disease when women and men were combined (HR = 1.41, 95% CI = 1.05–1.89, *P*-trend = 0.04) (**Table 3**). The positive association was stronger among men than women. Red meat and pork intakes were significantly and inversely associated with the risk of hemorrhagic stroke mortality (red meat: HR = 0.62, 95% CI = 0.45–0.87, *P*-trend = 0.02; pork: HR = 0.66, 95% CI = 0.47–0.91, *P*-trend = 0.08). These inverse associations were stronger among women than men. In contrast, there was no association between poultry intake and CVD-specific mortality.

**Table 3:** Risk of CVD-specific mortality by quintiles of meat intake.*

			Red meat				Poultry				Pork			
	Quintile		Deaths	HR	95%	CI	Deaths	HR	95%	CI	Deaths	HR	95%	CI
Women														
Ischemic heart														
disease	1		91	Reference			90	Reference			85	Reference		
	2		68	1.03	0.75,	1.43	62	0.94	0.67,	1.30	79	1.23	0.90,	1.68
	3		69	1.33	0.96,	1.86	65	1.34	0.96,	1.88	66	1.30	0.93,	1.82
	4		35	0.83	0.55,	1.26	51	1.29	0.89,	1.86	35	0.82	0.54,	1.24
	5		43	1.28	0.84,	1.96	38	1.24	0.82,	1.89	41	1.19	0.77,	1.82
	*P*-trend				0.43				0.17				0.90	
Ischemic stroke														
	1		124	Reference			114	Reference			110	Reference		
	2		68	0.79	0.58,	1.07	70	0.85	0.62,	1.15	78	0.99	0.74,	1.33
	3		44	0.64	0.44,	0.91	47	0.81	0.57,	1.16	43	0.69	0.48,	1.00
	4		45	0.81	0.56,	1.18	50	1.05	0.73,	1.50	52	1.01	0.71,	1.45
	5		39	0.84	0.55,	1.28	39	1.04	0.69,	1.56	37	0.87	0.57,	1.34
	*P*-trend				0.38				0.56				0.54	
Hemorrhagic														
stroke	1		111	Reference			87	Reference			107	Reference		
	2		67	0.76	0.56,	1.03	83	1.31	0.97,	1.79	65	0.75	0.55,	1.02
	3		52	0.68	0.48,	0.96	62	1.30	0.92,	1.83	51	0.68	0.48,	0.96
	4		51	0.76	0.53,	1.08	44	1.07	0.73,	1.58	61	0.90	0.64,	1.27
	5		37	0.57	0.37,	0.87	42	1.20	0.79,	1.80	34	0.52	0.34,	0.81
	*P*-trend				0.01				0.77				0.02	
Men														
Ischemic heart														
disease		1	84	Reference			74	Reference			83	Reference		
		2	64	1.04	0.75,	1.45	57	0.83	0.59,	1.18	61	0.97	0.69,	1.36
		3	43	0.88	0.60,	1.30	49	0.86	0.59,	1.26	44	0.85	0.58,	1.24
		4	40	0.97	0.65,	1.45	54	0.94	0.65,	1.36	41	0.94	0.63,	1.41
		5	53	1.54	1.02,	2.32	50	0.95	0.63,	1.41	55	1.45	0.97,	2.17
		*P*-trend			0.07				0.89				0.09	
Ischemic stroke														
		1	66	Reference			50	Reference			63	Reference		
		2	47	1.02	0.70,	1.50	46	1.01	0.67,	1.53	46	1.01	0.68,	1.49
		3	27	0.82	0.52,	1.31	35	0.99	0.63,	1.55	28	0.81	0.51,	1.28
		4	23	0.91	0.55,	1.51	29	0.86	0.53,	1.40	22	0.86	0.51,	1.45
		5	21	1.22	0.69,	2.15	24	0.92	0.54,	1.57	25	1.35	0.79,	2.31
		*P*-trend			0.73				0.63				0.50	
Hemorrhagic														
stroke		1	77	Reference			62	Reference			70	Reference		
		2	38	0.65	0.44,	0.96	50	0.88	0.60,	1.29	44	0.81	0.55,	1.19
		3	36	0.76	0.50,	1.15	37	0.79	0.52,	1.21	31	0.69	0.44,	1.06
		4	34	0.82	0.53,	1.27	27	0.59	0.37,	0.95	36	0.94	0.61,	1.44
		5	27	0.71	0.43,	1.20	36	0.89	0.56,	1.40	31	0.88	0.54,	1.44
		*P*-trend			0.32				0.52				0.74	
Women+Men†														
Ischemic heart														
disease		1	175	Reference			164	Reference			168	Reference		
		2	132	1.04	0.82,	1.31	119	0.89	0.70,	1.13	140	1.10	0.88,	1.39
		3	112	1.12	0.87,	1.44	114	1.10	0.86,	1.42	110	1.07	0.83,	1.38
		4	75	0.90	0.67,	1.20	105	1.10	0.84,	1.43	76	0.88	0.66,	1.17
		5	96	1.41	1.05,	1.89	88	1.08	0.81,	1.44	96	1.32	0.98,	1.77
		*P*-trend			0.04				0.33				0.20	
			*Q* = 0.35, *P*-heterogeneity = 0.55				*Q* = 0.84, *P*-heterogeneity = 0.36				*Q* = 0.45, *P*-heterogeneity = 0.50			
Ischemic stroke														
		1	190	Reference			164	Reference			173	Reference		
		2	115	0.87	0.69,	1.10	116	0.90	0.71,	1.15	124	1.00	0.79,	1.26
		3	71	0.70	0.53,	0.93	82	0.87	0.66,	1.16	71	0.73	0.55,	0.98
		4	68	0.84	0.63,	1.14	79	0.98	0.73,	1.30	74	0.96	0.72,	1.29
		5	60	0.96	0.68,	1.35	63	0.99	0.72,	1.37	62	1.03	0.74,	1.44
		*P*-trend			0.66				0.89				0.97	
			*Q* = 1.04, *P*-heterogeneity = 0.31				*Q* = 0.13, *P*-heterogeneity = 0.72				*Q* = 1.55, *P*-heterogeneity = 0.21			
Hemorrhagic														
stroke		1	188	Reference			149	Reference			177	Reference		
		2	105	0.71	0.56,	0.91	133	1.12	0.88,	1.42	109	0.77	0.61,	0.98
		3	88	0.71	0.54,	0.93	99	1.07	0.82,	1.39	82	0.68	0.52,	0.89
		4	85	0.78	0.59,	1.03	71	0.84	0.62,	1.14	97	0.91	0.70,	1.20
		5	64	0.62	0.45,	0.87	78	1.05	0.77,	1.42	65	0.66	0.47,	0.91
		*P*-trend			0.02				0.81				0.08	
			*Q* = 0.44, *P*-heterogeneity = 0.51				*Q* = 0.92, *P*-heterogeneity = 0.34				*Q* = 2.37, *P*-heterogeneity = 0.12			

* Adjusted for age at baseline, total caloric intake, income, occupation, education, comorbidity index, physical activity level, total vegetable intake, total fruit intake, fish intake, and red meat or poultry intake where appropriate, smoking history (ever/never smoking for women and pack-years of smoking for men), and alcohol consumption (for men only).

† Combined risk estimates derived from a random-effects meta-analysis are presented when the test for heterogeneity was significant; combined risk estimates with a fixed-effects meta-analysis are presented when significant heterogeneity was not present. *Q* statistics and *P*-heterogeneity for risk estimates comparing the highest quintiles of intake with the lowest are shown.

The association between red meat intake and lung cancer mortality differed between women and men (*P*-heterogeneity = 0.03), although neither the association among men (HR = 1.32, 95% CI = 0.90–1.94, *P*-trend = 0.12) nor the association among women (HR = 0.72, 95% CI = 0.48–1.07, *P*-trend = 0.25) were statistically significant (**Table 4**). There were no associations between red meat intake and death from other major cancers, or smoking-related or non-smoking related cancers (data not shown) among women or men. Poultry and pork intakes were not associated with any cancer-specific mortality. In addition, as a nutrient related to red meat intake and serum cholesterol, we observed inverse associations of saturated fat intake with ischemic stroke mortality among women and men and with hemorrhagic stroke mortality among women only; saturated fat intake had a positive association with liver cancer mortality among men (data not shown).

**Table 4:** Risk of cancer-specific mortality by quintiles of meat intake.*

			Red meat				Poultry				Pork			
	Quintile		Deaths	HR	95%	CI	Deaths	HR	95%	CI	Deaths	HR	95%	CI
Women														
Lung cancer														
		1	101	Reference			99	Reference			97	Reference		
		2	76	0.92	0.68,	1.25	87	1.00	0.75,	1.35	79	0.97	0.72,	1.32
		3	66	0.89	0.64,	1.23	55	0.77	0.54,	1.08	68	0.94	0.68,	1.30
		4	74	1.12	0.81,	1.55	58	0.87	0.62,	1.24	70	1.07	0.77,	1.49
		5	47	0.72	0.48,	1.07	65	1.11	0.78,	1.58	50	0.78	0.53,	1.15
		*P*-trend			0.25				0.52				0.32	
Stomach cancer														
		1	66	Reference			59	Reference			66	Reference		
		2	45	0.81	0.55,	1.20	44	0.89	0.60,	1.32	42	0.73	0.49,	1.08
		3	46	0.95	0.64,	1.42	56	1.40	0.95,	2.06	52	1.04	0.71,	1.52
		4	39	0.93	0.61,	1.43	40	1.11	0.72,	1.72	38	0.85	0.56,	1.31
		5	34	0.95	0.58,	1.53	31	1.05	0.65,	1.70	32	0.82	0.51,	1.33
		*P*-trend			0.99				0.75				0.60	
Colorectal														
cancer		1	67	Reference			56	Reference			68	Reference		
		2	58	0.99	0.69,	1.42	53	1.07	0.73,	1.57	58	0.94	0.66,	1.35
		3	44	0.80	0.54,	1.19	48	1.16	0.77,	1.73	44	0.77	0.52,	1.15
		4	51	1.00	0.67,	1.48	63	1.61	1.09,	2.37	50	0.92	0.62,	1.36
		5	41	0.83	0.53,	1.31	41	1.14	0.73,	1.78	41	0.78	0.50,	1.22
		*P*-trend			0.48				0.38				0.31	
Liver cancer														
		1	44	Reference			44	Reference			42	Reference		
		2	20	0.53	0.31,	0.91	22	0.55	0.33,	0.93	22	0.59	0.35,	0.99
		3	25	0.70	0.42,	1.18	23	0.69	0.41,	1.17	27	0.79	0.47,	1.30
		4	30	0.90	0.54,	1.50	25	0.78	0.46,	1.32	27	0.84	0.50,	1.41
		5	22	0.63	0.34,	1.14	27	0.89	0.52,	1.53	23	0.66	0.37,	1.19
		*P*-trend			0.39				0.73				0.37	
Men														
Lung cancer														
		1	104	Reference			95	Reference			109	Reference		
		2	73	0.98	0.72,	1.33	82	0.89	0.66,	1.20	68	0.84	0.62,	1.14
		3	68	1.14	0.83,	1.57	64	0.82	0.59,	1.14	60	0.89	0.64,	1.23
		4	59	1.15	0.82,	1.63	63	0.81	0.58,	1.13	66	1.17	0.84,	1.62
		5	56	1.32	0.90,	1.94	56	0.78	0.55,	1.13	57	1.14	0.79,	1.66
		*P*-trend			0.12				0.22				0.23	
Stomach cancer														
		1	44	Reference			38	Reference			39	Reference		
		2	29	0.79	0.49,	1.27	26	0.65	0.39,	1.08	27	0.81	0.49,	1.34
		3	30	0.91	0.56,	1.47	37	1.00	0.62,	1.60	33	1.12	0.69,	1.82
		4	30	0.96	0.58,	1.59	31	0.80	0.48,	1.32	30	1.09	0.66,	1.82
		5	32	1.08	0.63,	1.86	33	0.85	0.51,	1.42	36	1.38	0.81,	2.34
		*P*-trend			0.59				0.87				0.14	
Liver cancer														
		1	46	Reference			36	Reference			44	Reference		
		2	36	0.89	0.57,	1.38	26	0.70	0.42,	1.17	38	0.96	0.62,	1.49
		3	25	0.66	0.40,	1.09	38	1.14	0.71,	1.82	25	0.67	0.40,	1.11
		4	29	0.80	0.48,	1.32	34	1.00	0.61,	1.63	28	0.80	0.48,	1.32
		5	32	0.95	0.55,	1.62	34	1.03	0.62,	1.73	33	0.96	0.57,	1.63
		*P*-trend			0.86				0.57				0.82	
Colorectal														
cancer		1	31	Reference			26	Reference			32	Reference		
		2	27	1.10	0.65,	1.86	21	0.75	0.42,	1.34	24	0.92	0.54,	1.57
		3	26	1.21	0.70,	2.09	23	0.93	0.52,	1.66	28	1.21	0.71,	2.06
		4	16	0.84	0.44,	1.59	26	0.98	0.55,	1.75	17	0.81	0.43,	1.51
		5	20	1.11	0.57,	2.15	24	0.91	0.49,	1.68	19	0.92	0.48,	1.78
		*P*-trend			0.98				0.91				0.73	
Women+Men†														
Lung cancer														
		1	205	Reference			194	Reference			206	Reference		
		2	149	0.95	0.77,	1.18	169	0.95	0.77,	1.17	147	0.90	0.73,	1.12
		3	134	1.01	0.80,	1.27	119	0.79	0.63,	1.01	128	0.91	0.73,	1.15
		4	133	1.13	0.89,	1.44	121	0.84	0.66,	1.07	136	1.12	0.88,	1.41
		5	103	0.98	0.54,	1.77	121	0.94	0.73,	1.21	107	0.95	0.73,	1.25
		*P*-trend			0.71				0.68				>0.99	
			*Q* = 4.63, *P*-heterogeneity = 0.03				*Q* = 1.86, *P*-heterogeneity = 0.17				*Q* = 1.97, *P*-heterogeneity = 0.16			
Stomach cancer														
		1	110	Reference			97	Reference			105	Reference		
		2	74	0.80	0.60,	1.09	70	0.79	0.58,	1.08	69	0.76	0.56,	1.04
		3	76	0.93	0.69,	1.27	93	1.22	0.90,	1.64	85	1.07	0.79,	1.44
		4	69	0.94	0.68,	1.31	71	0.97	0.70,	1.34	68	0.95	0.68,	1.31
		5	66	1.00	0.70,	1.44	64	0.95	0.67,	1.35	68	1.04	0.72,	1.49
		*P*-trend			0.70				0.90				0.35	
			*Q* = 0.13, *P*-heterogeneity = 0.72				*Q* = 0.35, *P*-heterogeneity = 0.56				*Q* = 1.97, *P*-heterogeneity = 0.16			
Colorectal														
cancer		1	98	Reference			82	Reference			100	Reference		
		2	85	1.02	0.76,	1.38	74	0.96	0.70,	1.32	82	0.94	0.70,	1.26
		3	70	0.92	0.67,	1.27	71	1.08	0.77,	1.50	72	0.91	0.66,	1.24
		4	67	0.95	0.68,	1.33	89	1.38	1.00,	1.90	67	0.89	0.64,	1.24
		5	61	0.91	0.63,	1.32	65	1.05	0.73,	1.51	60	0.82	0.57,	1.19
		*P*-trend			0.52				0.47				0.38	
			*Q* = 0.49, *P*-heterogeneity = 0.48				*Q* = 0.32, *P*-heterogeneity = 0.57				*Q* = 0.11, *P*-heterogeneity = 0.74			
Liver cancer														
		1	90	Reference			80	Reference			86	Reference		
		2	56	0.72	0.51,	1.01	48	0.62	0.43,	0.90	60	0.78	0.56,	1.09
		3	50	0.68	0.47,	0.98	61	0.91	0.64,	1.30	52	0.73	0.51,	1.04
		4	59	0.85	0.59,	1.21	59	0.89	0.62,	1.27	55	0.82	0.57,	1.17
		5	54	0.79	0.53,	1.17	61	0.96	0.66,	1.40	56	0.81	0.55,	1.20
		*P*-trend			0.51				0.48				0.40	
			*Q* = 1.00, *P*-heterogeneity = 0.32				*Q* = 0.15, *P*-heterogeneity = 0.70				*Q* = 0.26, *P*-heterogeneity = 0.61			

* Adjusted for age at baseline, total caloric intake, income, occupation, education, comorbidity index, physical activity level, total vegetable intake, total fruit intake, fish intake, red meat intake, or poultry intake where appropriate, smoking history (ever/never smoking for women and pack-years of smoking for men), and alcohol consumption (for men only).

† Combined risk estimates derived from a random-effects meta-analysis are presented when the test for heterogeneity was significant; combined risk estimates from a fixed-effects meta-analysis are presented when significant heterogeneity was not present. *Q* statistics and *P*-heterogeneity for risk estimates comparing the highest quintiles of intake with the lowest are shown.

Among men, tests for non-linear associations with red meat intake were statistically significant for total and ischemic heart disease mortality (*P* = 0.01 and 0.03, respectively); non-linearity tests for other associations were not significant. Among women, none of the non-linear association tests were significant. When an energy density model was applied in the analyses, the overall results were materially unchanged, except for the following associations among men. Inverse associations of poultry intake with total and all-CVD mortality became statistically significant for the highest quintile of intake compared with the lowest (total mortality: HR = 0.86, 95% CI = 0.76–0.97; all-CVD mortality: HR = 0.78, 95% CI = 0.63–0.97), although the tests for linear trend were not significant (*P*-trend = 0.15 and 0.07). Positive associations of red meat intake with all-cancer mortality (HR = 1.25, 95% CI = 1.04–1.50; *P*-trend = 0.04) and lung cancer mortality (HR = 1.55, 95% CI = 1.11–2.14; *P*-trend = 0.03) became statistically significant. In contrast, the positive association between red meat intake and ischemic heart disease mortality lost statistical significance (HR = 1.30, 95% CI = 0.90–1.89; *P*-trend = 0.14).

Stratified analyses showed generally consistent patterns of associations across strata, except for the following associations with significant interaction effects (data not shown). Among women with a high educational level, red meat intake was positively, but not significantly, associated with all-CVD mortality, whereas among women with a low educational level, it was inversely and non-significantly associated with all-CVD mortality (*P*-interaction = 0.03). A similar interaction effect was observed for the association with hemorrhagic stroke mortality (*P*-interaction = 0.002). Among men, a significant interaction effect for income was observed in the association between red meat intake and total mortality (*P*-interaction = 0.0003) where there was a significant positive association in the low-income group and no association in the high-income group. There were no significant interaction effects for smoking status or regular alcohol consumption in associations between red meat intake and total or cause-specific mortality.

## Discussion

In our two large, population-based, cohort studies involving 134,290 middle-aged and elderly women and men in Shanghai, China, we found that red meat intake was positively associated with total mortality among men, but not among women. This discrepant association was also observed for lung cancer mortality. Further, red meat intake was positively associated with the risk of ischemic heart disease mortality, which was statistically significant among men. In contrast, red meat intake was inversely associated with the risk of hemorrhagic stroke mortality, which was statistically significant among women. Among men, the positive association between red meat intake and total mortality was significant in the low-income group, but no association was observed in the high-income group, with a significant interaction effect.

Two previous studies reported positive associations between red meat intake and total mortality among both men and women [1], [2]. Hence, our finding of positive associations between red meat intake and total mortality among men is consistent with these studies, whereas the null association observed among women conflicts with these studies. Our study is the first to report a sex difference in the effect of red meat intake on total mortality and this finding needs to be interpreted with caution. In our cohorts, the consumption of processed meat was very low and, hence, was not assessed quantitatively. Intake of unprocessed red meat in our population (median: 43 g/day among women and 54 g/day among men) is relatively lower than that reported in study populations in the United States (median: 49 g/day among women and 63 g/day among men in the National Institutes of Health-American Association of Retired Persons cohort; mean intake range: 94 g/day in 1980 to 47 g/day in 2006 among women in the Nurses’ Health Study; mean intake range: 64 g/day in 1986 to 54 g/day in 2006 among men in the Health Professionals Follow-up Study) [1], [2]. Furthermore, the major red meat consumed in North America is beef [10], whereas in Shanghai it is pork. Given the consistent positive associations of intake of unprocessed red meat, individual red meat items, and processed meat with total mortality that were reported in the two previous studies [1], [2], the difference in unprocessed and processed meat intakes is less likely to explain the discrepancy in the findings among women. Moreover, the iron content in meats consumed in China might be lower than that in other countries, as blood is often drained from meat before consumption. Hence, the potential adverse effect of meat consumption due to iron content (e.g., oxidative stress and DNA damage) may be minimal in our study populations. This may partially explain our finding of no association among women, whose prevalence of iron deficiency may be relatively high [22]. Since smoking and alcohol consumption rates among women (2.8% and 2.2%, respectively) differ substantially from those of the men in our study population (smoking: 69.6% and alcohol consumption: 33.7%) and from those in US populations, the sex-specific association needs to be evaluated further in other populations where gender differences in these lifestyle factors are less striking than ours.

In addition, the association between red meat intake and lung cancer mortality differed by sex in our study. When associations were investigated separately, we did not find a significant association among women or men, which is inconsistent with a recent meta-analysis of 34 observational studies of incident and fatal lung cancer [23]. Among men in our study, 86.9% of the lung cancer deaths occurred in smokers, whereas only 10.7% of lung cancer deaths among women occurred in smokers, since only 2.8% of the women in the SWHS had ever smoked. To further elucidate our finding, we examined associations of red meat intake with smoking-related or non-smoking-related cancer deaths and found no associations among women or men. Differences in etiology and risk factors have been postulated between smoking-related and non-smoking-related lung cancers, since squamous cell carcinoma is more common among smokers and adenocarcinoma is more common among never smokers [24]. Potential effects of sex hormones on lung cancer mortality among women [25] and on lung cancer risk and all-cancer mortality among men [26], [27] have also been reported. However, to our knowledge, there is no known biological mechanism to explain the observed sex difference. The meta-analysis reported consistent positive associations between red meat intake and lung cancer risk, regardless of smoking status, sex, and histologic subtype [23]. Therefore, the factors that might explain the observed sex difference need clarification. Similar to red meat and total mortality, our study is the first to report a sex difference in the association between red meat intake and lung cancer mortality, which warrants further investigation.

In our study, the interaction between red meat intake and income was significant for total mortality among men. To our knowledge, no previous study has reported this interaction effect and, hence, income might have served as a surrogate for other factors in our study. Among lifestyle factors, regular alcohol consumption was slightly more common (36.5% and 30.2% for the low- and high-income groups, respectively) and vitamin supplement use was less common in the low-income group (9.7% and 19.3% for the low- and high-income groups, respectively), compared with the high-income group. Moreover, BMI was significantly and inversely correlated with red meat intake in the low-income group (*r* = −0.05), but was non-significantly and positively correlated in the high-income group (*r* = 0.003). These differences and, potentially, other unmeasured factors might have contributed to the differential associations by income group. Nonetheless, this finding warrants further investigation.

The observed positive association between red meat intake and ischemic heart disease mortality is consistent with the Seventh-day Adventists study report of a positive association for beef intake among men, but not among women [3]. However, it conflicts with another study of meat intake and mortality [5], which reported inverse associations of ischemic heart disease mortality with intake of unprocessed or processed meats. The association was stronger for intake of processed meat compared with unprocessed meat among men [5]. Our study did not collect information on the amount of processed meat consumed, but did obtain information on the frequency; only 4.8% and 2.8% of the participants in the SWHS and SMHS, respectively, reported having consumed processed meat at least once a week. Thus, we were not able to assess the association for processed meat intake. Altogether, few studies have investigated associations of red meat intake with ischemic heart disease mortality and, hence, our results need to be replicated in other populations.

Our finding of an inverse association between red meat intake and hemorrhagic stroke mortality generally agrees with previous reports on total serum cholesterol among Koreans [28], animal product intake and death from intracerebral hemorrhage [4], and saturated fat and animal protein intakes and the risk of intraparenchymal hemorrhage in the United States [29]. It is also consistent with the known etiology of and risk factors for hemorrhagic stroke [30]. However, this finding is in contrast to a recent meta-analysis of 20 observational studies [31] that reported a positive association with the risk of incident stroke of all subtypes (no meta-analysis of hemorrhagic stroke was reported, since only one study had investigated this subtype of stroke and had reported no association [32]). Since this meta-analysis included only one study conducted in an Asian population [4], it may not reflect the association typically observed in this population [33]. Further, inconsistent findings between our and previous studies might be explained by different lifestyle or other factors associated with red meat consumption in each study population. In our study, women in the highest quintile of red meat intake tended to have higher education and were more likely to have taken vitamin supplements than those in the lowest quintile. Although we carefully adjusted for these known confounders, we cannot completely rule out the possibility of residual confounding. Alternatively, the protective effect of red meat on stroke in previous studies may have been driven by residual confounding due to health consciousness [3], [34].

For poultry, one previous study in Japan reported no association between poultry intake and stroke mortality [4], which is consistent with our finding of no association with either ischemic or hemorrhagic stroke mortality. Poultry may be consumed as a healthier alternative to red meat and, hence, may indicate health consciousness, as observed among Chinese immigrants to the United States [35]. To our knowledge, only a few studies have specifically investigated the association of poultry intake with stroke mortality; this association needs to be investigated in other populations.

Strengths of our study include the population-based, prospective study design and high response rates for both the initial recruitment and follow ups. Unlike the few previous studies that only assessed the frequency of intake [4], [5], dietary intake in our study was assessed quantitatively (both frequency and amount of consumption) and prospectively by validated FFQs that were developed specifically for the study population [13], [14]. For limitations of this study, our study population had very low intakes of beef and lamb and of processed meat; hence, we did not assess associations of these foods with mortality risk. The follow up for men was shorter than for women, which led to low statistical power for the evaluation of associations of meat intake with mortality. In addition, our analysis focused only on baseline intake, given the shorter follow-up time among men and the potential effect of reverse causality, although a previous study reported stronger associations with repeated measures of dietary intake over time [2]. It is possible that meat intake levels may have changed during the past several decades due to economic growth and social changes in our study population. Because we only analyzed the dietary intake information collected at the baseline survey, the meat intake assessed in this analysis may not reflect long-term intake. Nevertheless, intakes of red meat and poultry changed little between the baseline survey and the first follow-up surveys with a difference of less than 3 g/1,000 kcal between 1997 and 2008. On the other hand, changes in meat intakes prior to our study enrollment may also have taken place. For example, approximately half of SMHS participants reported no change in intakes of red meat at 5 years before the baseline survey and about 20% reported a considerable change in intake, although similar information was not available for the SWHS. Hence, dietary changes associated with economic growth and social changes may have already occurred before our study enrollment and the meat consumption pattern among middle-aged and elderly Chinese men and women may have stabilized over the last 10 years. It is noteworthy that the SWHS had very few smokers and regular alcohol drinkers, reflecting the very low prevalence of these lifestyle factors among women in China, while the proportions of smokers and regular drinkers were high among men. Hence, some of the observed sex differences could be driven by these differences in the two cohorts.

In conclusion, in our study populations, where the consumption of processed meat is uncommon and pork is the predominant red meat, we found a sex difference in the association between red meat intake and total and lung cancer mortality. Since smoking and alcohol consumption patterns differ considerably between women and men in our study populations, this finding warrants further investigation.
